# Low fT3/fT4 ratio as a proxy for muscle wasting in patients with advanced non-small cell lung cancer treated with pembrolizumab

**DOI:** 10.3389/fonc.2025.1635321

**Published:** 2025-08-27

**Authors:** Serena Eccher, Marco Sposito, Ilaria Trestini, Ilaria Mariangela Scaglione, Luca Pasqualin, Daniela Tregnago, Alice Avancini, Jessica Insolda, Linda Confortini, Alessandra Dodi, Alessio Stefani, Marco Cintoni, Isabella Sperduti, Maria Cristina Mele, Fotios Loupakis, Marcello Tiseo, Emilio Bria, Michele Milella, Sara Pilotto, Lorenzo Belluomini

**Affiliations:** ^1^ Section of Oncology, Department of Engineering for Innovation Medicine (DIMI), University of Verona School of Medicine and Verona University Hospital Trust, Verona, Italy; ^2^ Dietetic Service, University and Hospital Trust (AOUI) of Verona, Verona, Italy; ^3^ Department of Neurosciences, Biomedicine and Movement Sciences, University of Verona, Verona, Italy; ^4^ Department of Medicine and Surgery, Medical Oncology Unit, University Hospital of Parma, Parma, Italy; ^5^ Comprehensive Cancer Center, Medical Oncology Department, Fondazione Policlinico Universitario Agostino Gemelli IRCCS, Rome, Italy; ^6^ Department of Translational Medicine and Surgery, Clinical Nutrition Unit, Fondazione Policlinico Universitario Agostino Gemelli IRCCS & Research and Training Center in Human Nutrition, Università Cattolica del Sacro Cuore, Rome, Italy; ^7^ Biostatistics, IRCCS Regina Elena National Cancer Institute, Rome, Italy; ^8^ 3trees Healthcare, Viterbo, Italy; ^9^ Medical Oncology Unit, Ospedale Isola Tiberina - Gemelli Isola, Rome, Italy

**Keywords:** non-small cell lung cancer (NSCLC), muscle wasting, immune checkpoint inhibitors (ICIs), thyroid hormones, sarcopenia, body composition

## Abstract

**Background:**

Loss of skeletal muscle mass may serve as a valuable indicator of treatment efficacy and survival in individuals with lung cancer undergoing immunotherapy. This investigation sought to pinpoint accessible markers that could reflect the presence of muscle degradation.

**Methods:**

A retrospective study was conducted on patients with advanced non-small cell lung cancer (NSCLC) who received first-line pembrolizumab therapy from June 2018 to September 2021. Data collected included computed tomography (CT)-based body composition, clinical and radiological characteristics, along with thyroid function tests (free triiodothyronine [fT3] and free thyroxine [fT4]). Predictive factors were evaluated using multivariate logistic regression models.

**Results:**

Among 31 patients, muscle wasting was observed in 58.1%. Performance Status (PS) emerged as a strong predictor (p=0.005), and a significant link was also found between muscle depletion and fT3/fT4 ratio (p=0.0296). After adjusting for PS, the association with the hormone ratio remained suggestive though not statistically definitive (p=0.091). ROC curve analysis identified a threshold value of 2.84 for fT3/fT4 ratio, which best differentiated patients at higher versus lower risk of muscle loss. Notably, 77.3% of individuals with muscle wasting had a ratio below this cut-off, compared to only 14.3% of those with higher ratios (p=0.006). While no significant correlation was found between the hormone ratio and progression-free survival (PFS), a meaningful association with overall survival (OS) was observed (p=0.032).

**Conclusions:**

Despite the limited sample size, fT3/fT4 ratio appears to be a promising and accessible biomarker for identifying muscle wasting, which may be linked to diminished treatment response and shorter survival in patients with NSCLC.

## Introduction

Immune checkpoint inhibitors (ICIs) have transformed the therapeutic paradigm for advanced non-small cell lung cancer (NSCLC), yielding substantial clinical benefits. However, durable responses are observed in only a minority of patients, underscoring the critical need for predictive biomarkers to optimize patient selection and therapeutic outcomes (1).

Skeletal muscle wasting (SMW) is a multifactorial condition influenced by aberrant interactions among cytokines, endocrine signaling pathways, metabolic alterations, nutritional deficits, and reduced physical activity (2). SMW, related to inflammation and metabolic dysfunction, may impair immune response and reduce immunotherapy efficacy in NSCLC (2). Moreover, in NSCLC and other malignancies, SMW has emerged as a negative prognostic factor and a potential negative biomarker of immunotherapy response (3–5).

Interestingly, type 2 and 3 iodothyronine deiodinases (DIO2 and DIO3) are present in skeletal muscle, where thyroid hormone signaling plays a crucial role in muscle development, function, and regeneration (6). In this light, the ratio of free triiodothyronine (fT3) to free thyroxine (fT4) is an easily assessable and reliable indicator of deiodinases regulating active thyroid hormones. Exploratory studies have linked a low fT3/fT4 ratio with reduced muscle mass and SMW in several non-cancer conditions (7, 8). Moreover, the fT3/fT4 ratio has been found to correlate with survival outcomes in patients with solid tumors (colorectal cancer and renal cell carcinoma) treated with tyrosine kinase inhibitors (9, 10) and in patients with urothelial carcinoma treated with immunotherapy (11). fT3/fT4 ratio, strictly related to muscle metabolism and systemic inflammation (6, 12), may reflect metabolic status and immune system function and thus serve as a useful predictor of immunotherapy response in NSCLC. Based on this evidence, we hypothesized that the fT3/fT4 ratio could serve as an easily assessable surrogate marker of skeletal muscle status and an indirect predictor for response to immunotherapy in patients with advanced NSCLC. We therefore conducted a study to explore the relationship between fT3/fT4 ratio, SMW and clinical outcome in NSCLC patients treated with upfront pembrolizumab.

## Materials and methods

We retrospectively analyzed a cohort of consecutive patients with advanced NSCLC treated with first-line pembrolizumab monotherapy between June 2018 and September 2021, at three Italian oncology centers (University of Verona, Gemelli University Hospital and Parma University Hospital). Inclusion criteria were: age >18 years; histologically confirmed diagnosis of advanced and measurable disease according to Response Evaluation Criteria In Solid Tumors (RECIST) 1.1 criteria; PD-L1 expression ≥ 50% assessed by immunohistochemistry (IHC); prior administration of more than one dose of pembrolizumab as first-line treatment, based on clinical judgment, aligned with established guidelines and best clinical practices; availability of pre-treatment abdominal computed tomography (CT) scans, performed within 3 months before starting immunotherapy. Patients with incomplete clinical data or lacking adequate imaging were excluded.

The study was approved by the local Ethical Committee (Prot. 2193CESC, 2022) and it was conducted according to the ethical standards of Declaration of Helsinki.

The following clinical-radiological and laboratory features were collected: age, gender, Eastern Cooperative Oncology Group Performance Status (ECOG PS), smoking history, comorbidities, disease stage, baseline thyroid hormones levels (fT3 and fT4), weight, height, Body Mass Index (BMI) and data on routine pre-immunotherapy CT based body composition.

Body composition assessment was performed by measuring the cross-sectional areas of skeletal muscle and fat tissue using a single axial CT slice taken at the level of the third lumbar vertebra (L3), a region known to reliably reflect total body tissue distribution (3).

Image analysis at the L3 level was carried out using a freehand segmentation approach, which closely aligned with the principles of the Alberta protocol (an established method based on tissue attenuation thresholds) utilizing built-in functionalities of SliceOmatic software version 5.0 (TomoVision, 3280 Chemin Milletta, Magog, J1X 0R4, Canada), distinguishing muscle from visceral, subcutaneous and intermuscular adipose tissues using anatomic knowledge and tissue-specific Hounsfield unit (HU) ranges. Skeletal muscle area (SMA) was identified within an attenuation range of −29 to +150 HU, visceral adipose tissue (VAT) was identified between -150 and -50 HU, subcutaneous adipose tissue (SAT) and intermuscular adipose tissue (IMAT) between -190 and -30 HU. The evaluated muscle groups included the psoas, erector spinae, quadratus lumborum, transversus abdominis, internal and external obliques, and rectus abdominis. To account for individual body size, the SMA was normalized to body surface area, resulting in the skeletal muscle index (SMI), expressed in cm²/m². This step was essential to precisely quantify muscle volume, which can vary significantly depending on an individual’s body composition. To categorize patients exhibiting skeletal muscle wasting (SMW), we employed sex-specific and BMI-adjusted SMI thresholds as outlined by Martin et al (13).

SMW was defined as SMI of <43 cm^2^/m^2^ for men with BMI < 25 kg/m^2^, SMI < 53 cm^2^/m^2^ for men with BMI ≥ 25 kg/m^2^, and SMI < 41 cm^2^/m^2^ for women. The main objective of the study was the association of SMW with predictive factors (e.g., ECOG PS, fT3/fT4 ratio).

Continuous variables were expressed as medians and range, categorical variables as frequencies and percentages. Progression-free survival (PFS) and overall survival (OS) were estimated using Kaplan-Meier methods and compared using the log-rank test. A p-value < 0.05 was considered statistically significant. A multivariate logistic regression, including ECOG PS and fT3/fT4 ratio, was developed using stepwise regression (forward selection, enter limit and remove limit, p = 0.10 and p = 0.15, respectively), to identify independent predictors of outcome. Receiver operating characteristic (ROC) analysis was conducted to determine the optimal cut-off for the fT3/fT4 ratio in predicting SMW. Statistical evaluation was performed by use of the SPSS 29.0 (SPSS Inc., Chicago, IL, USA) for Windows.

## Results

### Patients’ characteristics

Thirty-one patients with advanced NSCLC who received first-line pembrolizumab were evaluated for clinical and metabolic parameters, as described above. Of the patients analyzed, 21 (67.7%) were male, and the median age was 69 years (range: 44–85 years). A substantial proportion (90%) had a history of smoking, and 38.7% were active smokers at the initiation of treatment. Adenocarcinoma was the predominant histological subtype, observed in 67.7% of cases. The prevalence of muscle wasting was 58.1%. Values of basal fT3, fT4 and their ratio, were collected and reported in **Table 1**.

**Table 1:** Clinical characteristics of the cohort (n=31).

Patients’ characteristics	Number of patients, n = 31 (%)
Age at diagnosis (years)	
Median (range)	69 (44-85)
Gender	
Male	21 (67.7)
Female	10 (32.3)
Smoke	
Current	12 (38.7)
Former	16 (51.6)
Never	2 (6.5)
Not available	1 (3.2)
PS ECOG	
0	11 (35.5)
1	11 (35.5)
2	9 (29.0)
Histology	
Adenocarcinoma	21 (67.7)
Squamous Cell Carcinoma	7 (22.6)
NOS	3 (9.7)
Stage	
IIIA	1 (3.2)
IIIB	2 (6.5)
IVA	11 (35.5)
IVB	17 (54.8)
Metastatic sites	
Lung and pleura	18 (58.1)
Lymph nodes	11 (35.5)
Bone	11 (35.5)
Liver	1 (3.2)
Brain	7 (22.6)
Other	6 (19.4)
fT3/fT4 ratio*	
High	7 (22.6)
Low	24 (77.4)
Skeletal muscle wasting	
Yes	18 (58.1)
No	11 (35.5)
NA	2 (6.5)
BMI	
Median (range)	24.6 (18,7-35,9)
SMA	
Median (range)	132,5 (75,2-197,2)
SMI	
Median (range)	43,7 (26,3-64,5)
IMAT	
Median (range)	14,1 (4,5-44,0)
VAT	
Median (range)	128,1 (11,8-344,4)
SAT	
Median (range)	156,8 (57,5-436,9)
SMR	
Median (range)	30,7 (18,6-56,5)

PS, performance status; NOS, not otherwise specified; NA, Not Available; BMI, Body Mass Index; IMAT, intermuscular adipose tissue; SAT, subcutaneous adipose tissue; SMA, skeletal muscle area; SMI, skeletal muscle index; SMR, skeletal muscle radiodensity; VAT, visceraladipose tissue. *2.84 as ratio cut-off identified by ROC analysis.

### Association between SMW and clinical variables

To investigate potential associations between SMW and other clinical variables, a regression model was applied. The analysis identified a statistically significant association between SMW and ECOG PS (p = 0.005). Interestingly, a significant association was observed between SMW and the fT3/fT4 ratio (p = 0.0296). When ECOG PS was accounted for as a confounding variable, the association between the fT3/fT4 ratio and muscle wasting demonstrated a trend toward significance (p = 0.091).

### fT3/fT4 ratio threshold, subgroup and survival analyses

**Figure 1:**
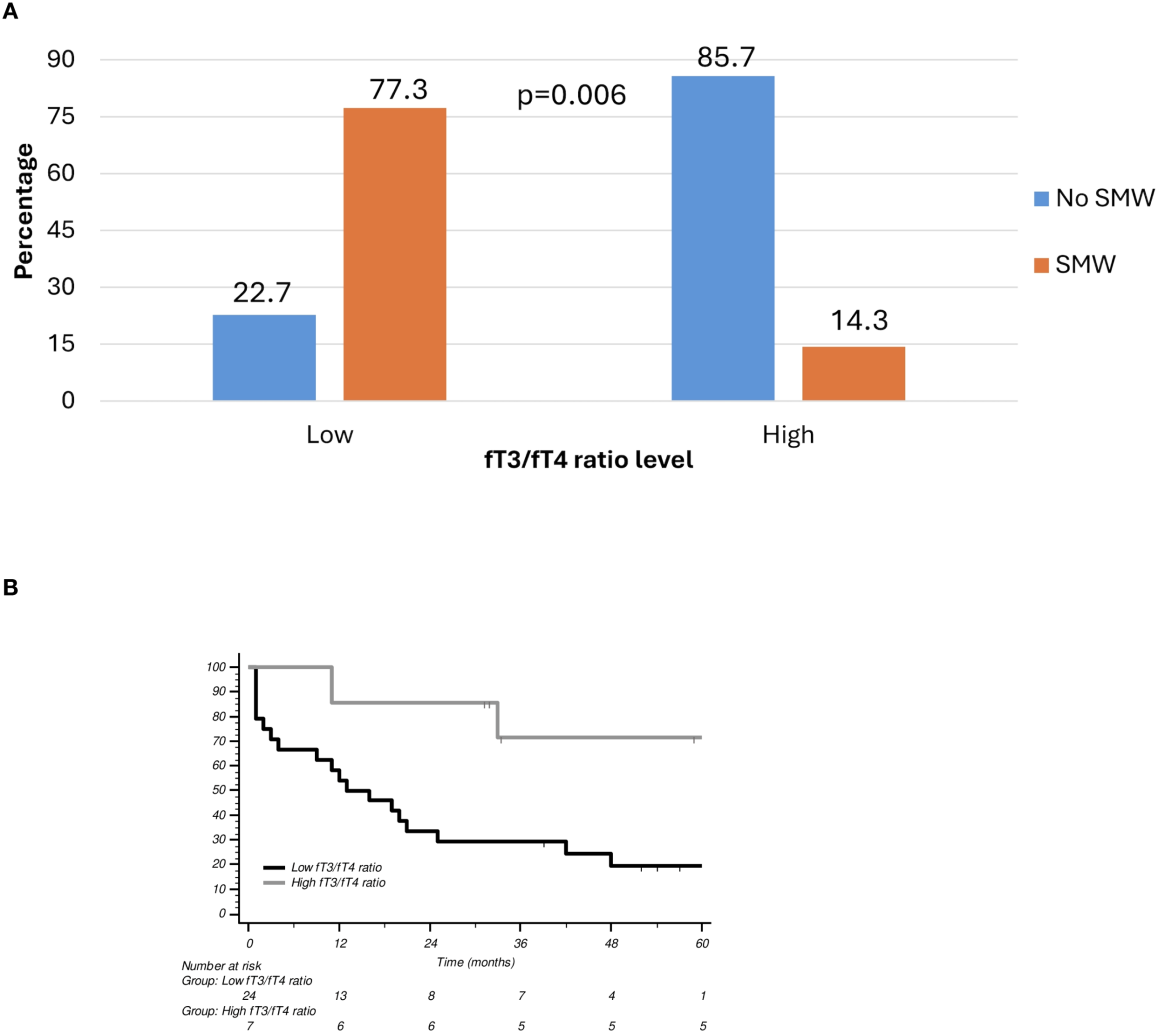
**(A)** Skeletal muscle wasting according to fT3/fT4 ratio level. **(B)** Overall survival (OS) curves according to fT3/fT4 ratio. SMW, Skeletal Muscle Wasting.

To further explore this relationship, ROC analysis was performed, which identified 2.84 as the optimal fT3/fT4 ratio threshold for distinguishing between low- and high-risk classifications for muscle depletion with the highest predictive accuracy. The ROC curve is a graphical plot that illustrates the diagnostic ability of a binary classifier system by plotting the true positive rate (sensitivity) against the false positive rate (1-specificity) at various threshold settings. In this context, the ROC curve was used to determine how well the fT3/fT4 ratio could discriminate between patients with and without muscle wasting. At the threshold of 2.84, the fT3/fT4 ratio demonstrated the highest predictive performance for identifying patients at risk of muscle depletion. This cut-off was then used to stratify patients into two groups: those with a “low” ratio (<2.84) and those with a “high” ratio (≥2.84). A subgroup analysis of patients with SMW revealed a marked disparity in the distribution of the fT3/fT4 ratio. Among these patients, 77.3% exhibited a low fT3/fT4 ratio, whereas only 14.3% had a high ratio, a statistically significant difference (p = 0.006) (**Figure 1A**). While no significant association was found between the fT3/fT4 ratio and PFS (p = 0.567), a significant correlation was observed with OS (p = 0.032) (**Figure 1B**).

## Discussion

Early identification, through clinical (and biological) biomarkers, of long-term responders to immunotherapy is essential for optimizing treatment strategies and improving clinical outcomes.

In our study, low fT3/fT4 ratio was significantly associated with a higher prevalence of SMW, even after adjusting for ECOG PS, preliminarily suggesting that fT3/fT4 ratio may serve as a proxy identifying SMW in patients with advanced NSCLC. Accordingly, recent findings in euthyroid individuals with type 2 diabetes mellitus demonstrated that a low fT3/fT4 ratio was significantly associated with sarcopenia, muscle mass, strength, and physical performance (7). Moreover, a significant inverse association between fT3/fT4 ratio and SMI (even after adjusting for age, metabolic factors, and comorbidities) was evaluated in a large retrospective study across Chinese subjects over 45 years old without cancer (8). In this light, fT3/fT4 ratio may reflect not only metabolic resilience but also systemic alterations associated with disease burden and catabolism, highlighting its potential role in identifying patients at increased risk of SMW and enabling earlier intervention and tailored supportive strategies in lung cancer.

Early detection of SMW is particularly relevant, representing one of the few ideally modifiable prognostic factors in several malignancies, including NSCLC (3). The baseline identification of these patients could facilitate their prompt referral to dedicated, multidisciplinary therapeutic interventions, such as tailored nutritional counselling, personalized exercise programs, and pharmacological approach, aimed at optimizing body composition and thus improving overall prognosis. Furthermore, while no statistically significant correlation was observed between the fT3/fT4 ratio and PFS (probably due to the small sample size), a significant association was identified with OS, suggesting a potential prognostic role for this parameter in immunotherapy-treated NSCLC.

However, our study has some limitations. Firstly, the small sample size may limit the statistical power and generalizability of our findings. Secondly, the exclusive inclusion of patients treated with pembrolizumab monotherapy may restrict the applicability of the results to broader NSCLC populations receiving other immune checkpoint inhibitors or chemoimmunotherapy combinations. Lastly, we assessed only baseline fT3/fT4 ratio values, without evaluating their longitudinal changes over the course of treatment, which could provide further insight into the dynamic relationship between thyroid function, treatment response and clinical outcomes. These limitations, along with our preliminary findings, have prompted us to design a prospective observational study to further investigate the role of the fT3/fT4 ratio in relation to body composition, as well as muscle strength and physical performance, and treatment response in advanced NSCLC. The study will specifically include patients undergoing immunotherapy, either alone or in combination with chemotherapy, to strengthen the clinical applicability of fT3/fT4 ratio. Furthermore, we will monitor longitudinal changes in fT3/fT4 ratio during treatment to evaluate its dynamic association with disease progression, therapeutic response, and muscle mass changes.

## Conclusions

fT3/fT4 ratio may represent a simple but reliable proxy for early detection of SMW in NSCLC patients receiving upfront mono-immunotherapy. Given the prognostic relevance of SMW, this parameter could support baseline clinical risk stratification. Further prospective studies are warranted to validate its utility and explore its association with immunotherapy response and long-term outcomes.

## Data Availability

The original contributions presented in the study are included in the article, further inquiries can be directed to the corresponding author/s.
